# Fitness advantages conferred by the L20-interacting RNA *cis*-regulator of ribosomal protein synthesis in *Bacillus subtilis*

**DOI:** 10.1261/rna.065011.117

**Published:** 2018-09

**Authors:** Arianne M. Babina, Darren J. Parker, Gene-Wei Li, Michelle M. Meyer

**Affiliations:** 1 Department of Biology, Boston College, Chestnut Hill, Massachusetts 02467, USA; 2 Department of Biology, Massachusetts Institute of Technology, Cambridge, Massachusetts 02139, USA

**Keywords:** RNA *cis*-regulator, gene regulation, ribosome assembly, rRNA, ribosomal protein, fitness

## Abstract

In many bacteria, ribosomal proteins autogenously repress their own expression by interacting with RNA structures typically located in the 5′-UTRs of their mRNA transcripts. This regulation is necessary to maintain a balance between ribosomal proteins and rRNA to ensure proper ribosome production. Despite advances in noncoding RNA discovery and validation of RNA-protein regulatory interactions, the selective pressures that govern the formation and maintenance of such RNA *cis*-regulators in the context of an organism remain largely undetermined. To examine the impact disruptions to this regulation have on bacterial fitness, we introduced point mutations that abolish ribosomal protein binding and regulation into the RNA structure that controls expression of ribosomal proteins L20 and L35 within the *Bacillus subtilis* genome. Our studies indicate that removing this regulation results in reduced log phase growth, improper rRNA maturation, and the accumulation of a kinetically trapped or misassembled ribosomal particle at low temperatures, suggesting defects in ribosome synthesis. Such work emphasizes the important role regulatory RNAs play in the stoichiometric production of ribosomal components for proper ribosome composition and overall organism viability and reinforces the potential of targeting ribosomal protein production and ribosome assembly with novel antimicrobials.

## INTRODUCTION

The ribosome is a complex molecular machine that plays an essential role in protein biosynthesis in all living organisms. The prokaryotic 70S ribosome consists of two subunits and is composed of three different rRNAs and over 50 different ribosomal proteins (for review, see Kaczanowska and Ryden-Aulin 2007; Shajani et al. 2011). In rapidly dividing *Escherichia coli*, as much as 40% of total cellular dry mass is comprised of ribosomes and their associated cofactors (Tissières et al. 1959; Nomura et al. 1984; Bremer and Dennis 2008), and ribosome production consumes ∼40% of the cell's total energy (Nierhaus 1991). Because of this enormous energy expense and the importance of appropriate component stoichiometry in ribosome synthesis and function, the production of individual ribosomal components and extraribosomal cofactors is highly regulated and tightly coordinated (Harvey 1970; Bremer and Dennis 2008).

One manner in which bacteria maintain the delicate balance between rRNA, ribosomal proteins, and extraribosomal cofactors to ensure proper and efficient ribosome production is through the autogenous regulation of ribosomal protein synthesis (Nomura et al. 1980, 1984; Nomura 1999). When rRNA-binding sites are saturated, several ribosomal proteins repress their own expression at the transcriptional or translational level by interacting with RNA structures typically found in the 5′-UTRs of their mRNA transcripts. This RNA-based negative-feedback regulation has been extensively characterized in the model gram-negative bacterium, *E. coli*. Fifteen distinct RNA *cis*-regulatory elements have been found to control the expression of over half of the ribosomal proteins within this organism (Zengel and Lindahl 1994; Fu et al. 2013; Matelska et al. 2013; Aseev et al. 2015, 2016). Four of these regulators are widely conserved across most bacterial phyla, while the remaining RNA structures are only found in Gammaproteobacteria (Iben and Draper 2008; Fu et al. 2013, 2014; Matelska et al. 2013). Nearly all bacterial phyla possess at least one of the widely distributed *cis*-regulatory RNA structures (Fu et al. 2013), setting the precedent for RNA-mediated autogenous regulation of ribosomal protein synthesis throughout bacteria. Furthermore, RNA structures that perform analogous regulatory functions in response to homologous proteins, yet share little to no similarity to the regulatory RNAs in *E. coli*, have been identified in different bacterial phyla (Grundy and Henkin 1991, 1992; Scott and Williamson 2001; Serganov et al. 2003; Choonee et al. 2007; Deiorio-Haggar et al. 2013; Slinger et al. 2014). These distinct regulatory RNA structures are typically narrowly distributed to specific bacterial groups and are likely the result of multiple instances of independent evolution. The presence of both highly conserved and independently derived *cis*-regulatory RNA structures suggests there exists significant selective pressure to regulate ribosomal protein synthesis in this manner.

Since the late 1970s, numerous studies have characterized the functional and structural nature of RNA-ribosomal protein regulatory interactions. However, our understanding of the selective pressures that drive the emergence and maintenance of RNA *cis*-regulators of ribosomal protein synthesis within bacterial genomes remains limited. Additionally, although it is well established that overexpressing autoregulatory ribosomal proteins inhibits bacterial growth, these observations are typically from studies that induced protein overexpression using multiple gene copies, plasmids, and/or strong promoters (Lindahl and Zengel 1979; Dean and Nomura 1980; Sykes et al. 2010). Little to no work has examined the effects of simply removing the autogenous regulation of ribosomal proteins in bacteria. To measure the fitness advantages ribosomal protein regulatory RNA structures confer to an organism, we introduced point mutations into the native locus of the previously characterized L20-interacting RNA *cis*-regulator in the *Bacillus subtilis* genome to disrupt ribosomal protein binding and regulation, and subsequently assayed the strains for mutant phenotypes.

We find that removing RNA-mediated autoregulation results in elevated transcript levels of downstream genes and causes cold-sensitive defects in growth, rRNA processing, and ribosomal subunit sedimentation. Our results suggest that improper regulation of ribosomal protein expression compromises ribosome biosynthesis and demonstrate the significant role *cis*-regulatory RNA structures have in proper ribosome production and overall organism fitness. This work gives insight into why RNA-based regulation of ribosomal proteins is so prevalent across diverse bacterial species and sheds light on the selective forces that govern structured RNA evolution and conservation.

## RESULTS

### Reporter assays confirm behavior of L20-interacting RNA mutations

Only two ribosomal protein RNA *cis*-regulators, the RNA structures interacting with L20 and the L10(L12)_4_ complex, have been mechanistically characterized in *B. subtilis* (Choonee et al. 2007; Bruscella et al. 2011; Yakhnin et al. 2015). The L20-interacting RNA structure found in *B. subtilis* is narrowly distributed to the Firmicutes phylum, and it regulates expression of the *infC-rpmI-rplT* operon (encoding translation initiation factor IF3 and large subunit ribosomal proteins L35 and L20, respectively) at the transcriptional level via a rho-independent terminator that is stabilized upon L20 binding (Choonee et al. 2007; Deiorio-Haggar et al. 2013). The structure, sites of RNA-protein interaction, and regulatory mechanism of the *B. subtilis* L20-interacting RNA are well characterized, rendering it a good subject for this study (Choonee et al. 2007; Bruscella et al. 2011).

**FIGURE 1.**
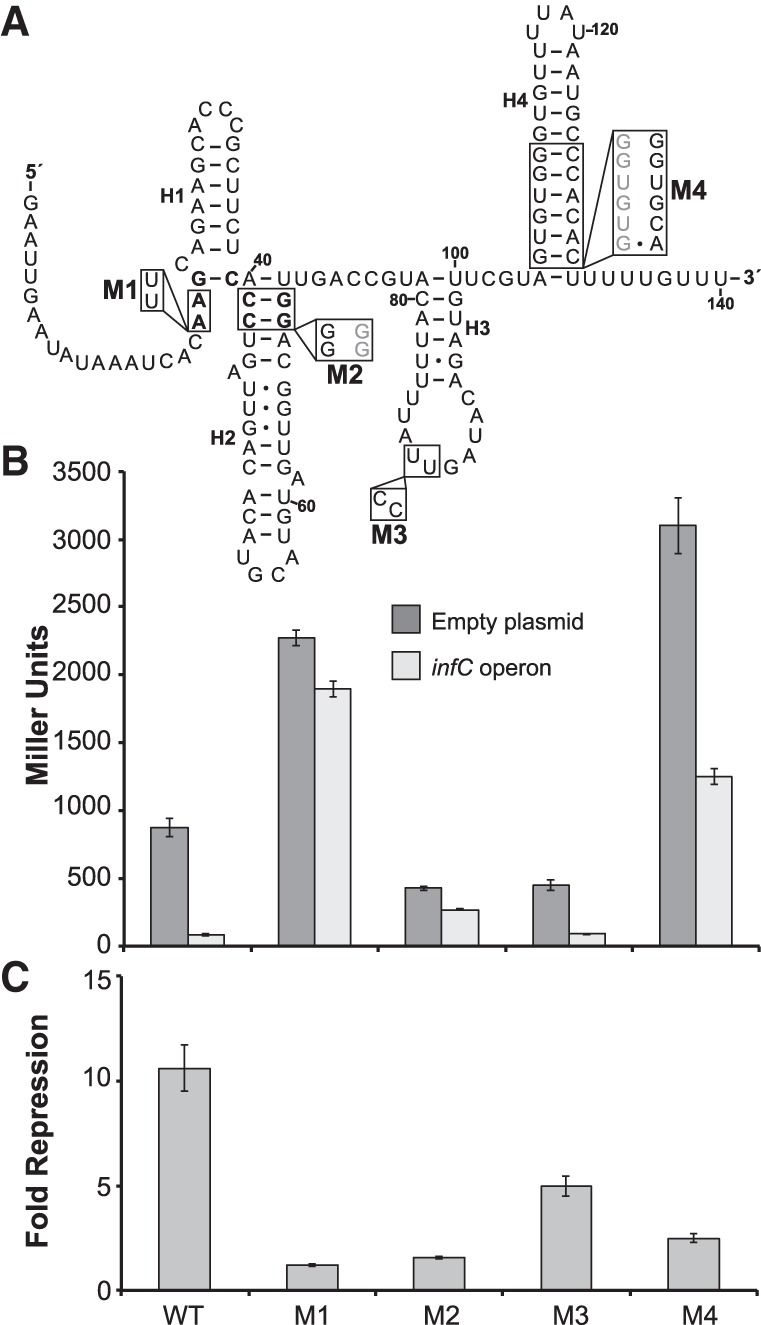
Regulatory activity of the *B. subtilis* L20-interacting RNA mutants examined in this study. (*A*) Secondary structure of the L20-interacting RNA in its protein-bound form with mutations M1–M4. The L20-binding site is in bold. Helix H4 is the rho-independent transcription terminator that forms upon L20 binding. Nucleotides are numbered from the transcript start site from the second *infC* operon promoter, +1 (Choonee et al. 2007). (*B*) β-galactosidase activity (in Miller Units) of the L20-interacting RNA mutant constructs with overexpression of the *infC* operon or empty plasmid during log phase growth at 37°C. The values reported represent the mean of three or more independent experimental replicates; error bars represent standard error of the mean across biological replicates. (*C*) Fold repression of the L20-interacting RNA reporter constructs derived from the data in *B*. Fold repression was calculated for each reporter construct as follows: (Mean Miller Units of empty plasmid strain)/(Mean Miller Units of *infC* operon overexpression strain). Error bars represent standard error of the mean propagated from the values in *B* using standard calculations (Taylor 1997).

We first verified the impact of specific mutations on the regulatory activity of the L20-interacting RNA and downstream gene expression using β-galactosidase reporter assays in *B. subtilis* (Fig. 1A). Assays were conducted during log phase growth while overexpressing the entire *infC* operon from a plasmid, or with an empty plasmid. An ∼11-fold reduction in β-galactosidase activity was observed for the wild-type L20-interacting RNA construct when the *infC* operon was overexpressed (Fig. 1B,C). This fold repression is comparable to what has been previously measured for the L20-interacting RNA (Choonee et al. 2007; Bruscella et al. 2011).

Mutations M1 and M2 disrupt the previously identified and highly conserved L20-binding site at the junction of Helices H1 and H2 (Fig. 1A; Choonee et al. 2007; Deiorio-Haggar et al. 2013). Both mutations relieve the repression observed with the wild-type RNA construct when the *infC* operon is overexpressed (Fig. 1B,C). Mutation M3 targets the loop region of Helix H3 and serves as a control. Previous nuclease probing analyses suggest that Helix H3 is not involved in L20-binding interactions (Choonee et al. 2007), and this stem is only present in ∼75% of examples of this RNA (Deiorio-Haggar et al. 2013). As anticipated, regulation was retained for the M3 mutant construct (approximately fivefold repression) (Fig. 1B,C).

Finally, the M4 mutation is designed to destabilize the stem of the rho-independent terminator that forms upon L20 binding (Fig. 1A). This mutation resulted in elevated constitutive expression. The activity measured for the M4 mutant under both unrepressed (empty plasmid) and repressed (*infC* operon overexpression) conditions was substantially higher than that obtained with the wild-type RNA (Fig. 1B,C). Regulatory activity with the M4 mutant RNA was substantially reduced (∼2.5-fold repression upon *infC* operon overexpression).

### L20-interacting RNA mutant recombinant strains are cold-sensitive

**FIGURE 2.**
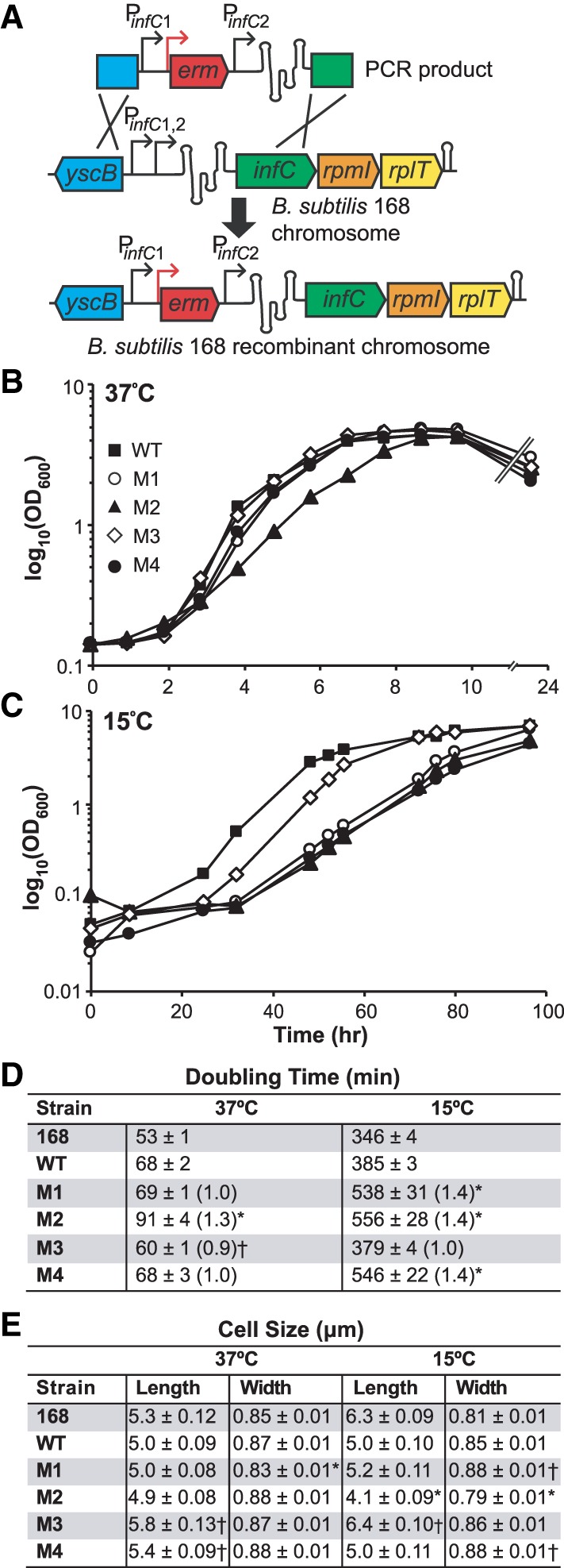
Construction and growth of L20-interacting RNA native locus recombinant strains. (*A*) L20-interacting RNA recombinant strain design. The second *infC* operon promoter (P_*infC*2_), L20-interacting regulatory RNA sequence, and two ∼500 bp regions flanking the promoter and regulatory RNA locus were PCR-amplified from *B. subtilis* 168 genomic DNA. A PCR product in which an erythromycin resistance cassette (*erm*) was introduced immediately upstream of the second *infC* operon promoter was generated, subcloned, and transformed into *B. subtilis* 168. Integration of the PCR constructs at the *infC* locus via double-crossover homologous recombination replaced the native L20-interacting RNA with a wild-type or mutant recombinant version. Growth curves for each recombinant strain in 2XYT at 37°C (*B*) and 15°C (*C*). Growth assays were performed three or more times for each strain. Representative curves are shown. (*D*) Doubling times (min) of L20-interacting RNA recombinant strains grown in 2XYT at 37°C and 15°C. Values were calculated from log phase OD_600_ values and are the mean of three or more independent experimental replicates; ± indicates the standard error of the mean across biological replicates. Numbers in parentheses denote mutant recombinant strain doubling time relative to that of the wild-type recombinant (WT) strain at the corresponding temperature. Asterisks (*) indicate mutant recombinant strains that grew significantly slower than the wild-type recombinant at the corresponding temperature. Daggers (†) indicate mutant recombinant strains that grew significantly faster than the wild-type recombinant at the corresponding temperature (*P* < 0.05). (*E*) L20-interacting RNA recombinant strain cell size (µm). Strains were grown to log phase (OD_600_ ∼ 0.4–0.5) in 2XYT at 37°C and 15°C and imaged using DIC microscopy. The lengths and widths of 100 cells from each strain were measured and averaged at each temperature; ± indicates the standard error of the mean. Asterisks (*) indicate mutant recombinant strains that were significantly smaller than the wild-type recombinant at the corresponding temperature. Daggers (†) indicate mutant recombinant strains that were significantly larger than the wild-type recombinant at the corresponding temperature (*P* < 0.05).

To investigate the effects mutations to the L20-interacting RNA and the loss of *infC* ribosomal protein operon regulation have on *B. subtilis* fitness, we replaced the native copy of the L20-interacting RNA within the *B. subtilis* 168 genome with either a wild-type or mutant recombinant version via homologous recombination (Fig. 2A). Growth curves (cell density measured by OD_600_) were performed to measure recombinant strain fitness, as global translation capacity and consequently ribosome quality and quantity can be inferred from log phase growth (Harvey 1970; Scott et al. 2014). Strains were assayed in nutrient-rich 2XYT medium at both 37°C and 15°C because sensitivity to low temperatures can be indicative of defects in ribosome assembly, composition, and/or function (Guthrie et al. 1969; Tai et al. 1969; Feunteun et al. 1974a; Isono et al. 1976, 1977; Charollais et al. 2004; Bharat and Brown 2014; Choudhury and Flower 2015).

The M1, M3, and M4 mutant recombinant strains grew similarly to, if not better than, the wild-type recombinant strain at 37°C, with doubling times of 69, 60, and 68 min, respectively, in comparison to the 68 min doubling time of the wild-type recombinant strain (Fig. 2B,D). The M2 protein-binding mutant recombinant strain grew approximately 1.3 times slower than the wild-type recombinant at 37°C, with a doubling time of 91 min. The M2 regulatory mutant recombinant strain was consistently unstable and difficult to maintain. While it is possible that additional mutations elsewhere in the genome contribute to the instability and slow growth exhibited by this strain, this growth defect at 37°C was observed for multiple M2 mutant recombinant strains generated from independent transformation events.

In contrast, all three mutant recombinant strains in which the regulatory activity of the L20-interacting RNA was compromised (M1, M2, M4) grew approximately 1.4 times slower than the wild-type recombinant at 15°C, whereas the M3 control mutant recombinant strain grew comparably to the wild-type recombinant at this temperature (Fig. 2C,D). This significant cold temperature-sensitive growth defect suggests that improper *infC* operon regulation likely affects ribosome production.

The 168 parental strain did not demonstrate any cold temperature-sensitive growth defects; rather, it consistently grew faster than the wild-type recombinant strain at both 37°C and 15°C. This difference is likely due to the presence of the erythromycin resistance cassette within the wild-type recombinant strain and/or the use of antibiotic in the recombinant strain medium. Consequently, the doubling times of the 168 parental strain are included in Figure 2D for reference, but the strain is not shown in the representative growth curves.

To assess whether OD_600_ measurements and subsequent doubling times were influenced by differences in cell size between recombinant strains, we measured the length and width of 100 cells of each strain grown to log phase in 2XYT medium at both 37°C and 15°C (Fig. 2E). Our measurements are consistent with those previously reported for log phase *B. subtilis* cells (Sargent 1975; Henriques et al. 1998; Sharpe and Errington 1998; Weart et al. 2007). We find that there is no correlation between cell size and the doubling times calculated from OD_600_ values at both temperatures. It is possible that the growth defect observed for the M2 regulatory mutant recombinant strain at 15°C is affected by the strain's significantly smaller cell size at this temperature. However, the M1 and M4 recombinant strains did not exhibit a cell size defect at 15°C, and all three regulatory mutant recombinant strains grew similarly at this temperature (1.4 times slower than the wild-type recombinant strain). The aforementioned instability of the M2 recombinant strain may contribute to the strain's reduced cell size at 15°C in some way. Overall, the slower doubling times calculated for the M1, M2, and M4 regulatory mutant recombinant strains at 15°C are likely due to cold temperature-sensitive growth defects rather than differences in cell size.

### Position of erythromycin resistance cassette does not influence recombinant strain phenotype

Following construction of our recombinant strains, we noticed that we failed to properly incorporate the first *infC* operon promoter into our native locus recombinant strain design. In *B. subtilis*, the *infC* operon is under the control of two promoters. Transcription from the first promoter adds 58 nucleotides to the 5′ end of the *infC* operon transcript, which contains an RNase Y cleavage site that ultimately allows for increased expression of ribosomal proteins L35 and L20 relative to IF3 (Bruscella et al. 2011). In our original recombinant strain design, we introduced the erythromycin resistance cassette immediately upstream of the second *infC* operon promoter, displacing the position of the first *infC* operon promoter (Fig. 2A). To determine whether disrupting the context of the two promoters influenced our results, we redesigned our wild-type recombinant and M1 mutant recombinant strains to more accurately reflect the native organization of the *infC* operon promoter region within the *B. subtilis* genome. In the re-designed recombinant strains, we inserted the erythromycin resistance cassette into the intergenic region immediately upstream of the first *infC* operon promoter (Supplemental Fig. S1A,B). Consistent with our previous findings, the re-designed M1 mutant recombinant strain exhibited cold-sensitive growth defects similar to that of the original M1 mutant recombinant strain (Supplemental Fig. S1D–F). Therefore, we continued to use the original recombinant strains we constructed for all subsequent experiments (Fig. 2A).

### Low temperature exacerbates *infC* operon misregulation at stationary phase in mutant recombinant strains

To further assess the origins of the temperature-sensitive phenotype observed during the growth assays, we quantified levels of the native *infC* operon transcript in the recombinant strains during both log (OD_600_ ∼ 0.3–0.7) and early stationary phase (OD_600_ ∼ 2.0–3.0) at 37°C and 15°C in the presence of endogenous L20 protein only. Because the L20-interacting RNA utilizes a transcription termination mechanism to regulate *infC* operon expression, we measured transcript levels using quantitative RT-PCR (qRT-PCR) with primers targeting the *rplT* (L20) coding region. While these measurements cannot capture the instantaneous rates of transcription or translation, the relative levels of *rplT* transcript do give some indication of whether differences in termination occur under these conditions, and whether there are likely to be differences in protein levels.

**FIGURE 3.**
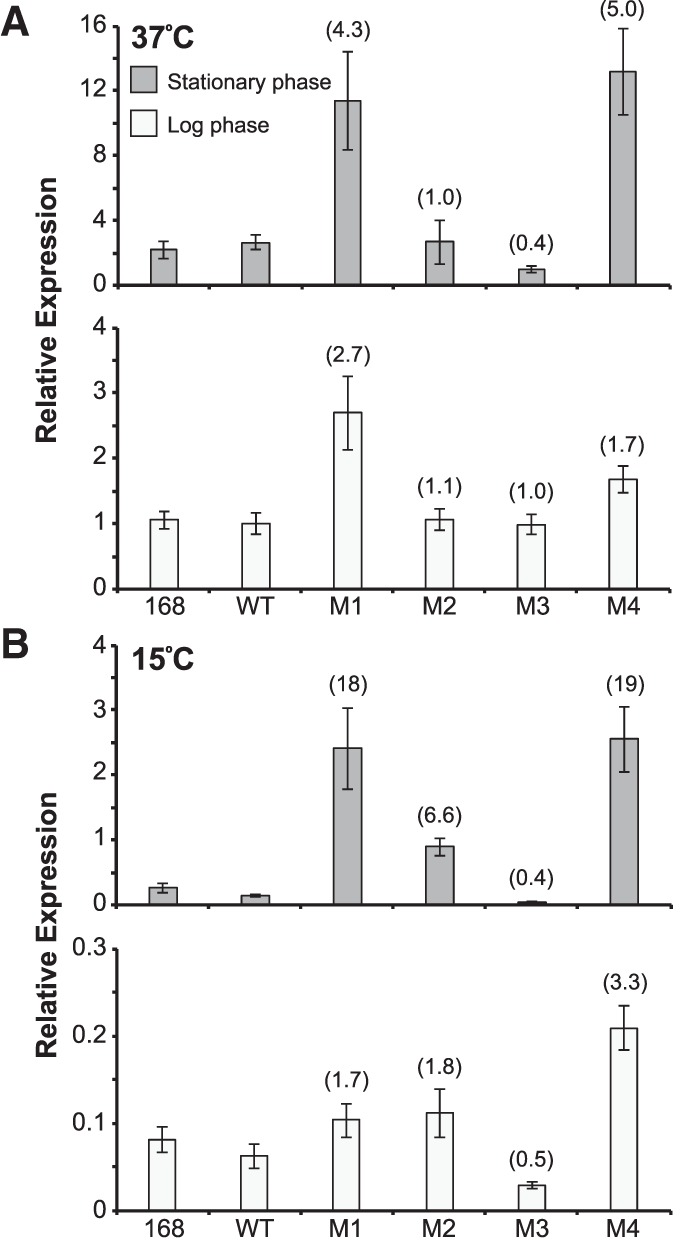
Recombinant strain *infC* operon transcript levels. qRT-PCR quantification of the native *rplT* transcript in each of the recombinant strains grown to log and early stationary phase at 37°C (*A*) and 15°C (*B*). For each strain/condition, *rplT* transcript level was normalized to the level of housekeeping gene *nifU* (Reiter et al. 2011). Graphs depict relative *rplT* transcript levels in each strain compared to the *rplT* level in the wild-type recombinant (WT) strain grown to log phase at 37°C. Error bars represent the standard error of the mean across three technical replicates. Numbers in parentheses denote mutant recombinant strain *rplT* transcript level relative to that of the wild-type recombinant strain at the corresponding growth phase and temperature.

The M2 and M3 mutant recombinant strains exhibited *rplT* transcript levels comparable to that of the wild-type recombinant during log phase at 37°C, while the M1 and M4 mutant recombinant strains demonstrated a ∼2.7 and 1.7-fold increase in *rplT* transcript, respectively, in comparison to that of the wild-type recombinant strain under these conditions (Fig. 3A). During early stationary phase at 37°C, *rplT* transcript levels in all strains were approximately twofold higher relative to the *nifU* internal control compared to log phase, and the increased transcript levels measured in the M1 and M4 mutant recombinant strains were even more prominent (∼4.3 and fivefold increase in *rplT* levels, respectively, compared to the wild-type recombinant). Under these conditions, *rplT* transcript in the M2 mutant recombinant strain remained consistent with that in the wild-type recombinant, and the M3 control mutant recombinant strain displayed a ∼2.5-fold decrease in *rplT* levels relative to the wild-type recombinant strain. The β-galactosidase activities measured for each L20-interacting RNA reporter in the absence of *infC* operon overexpression (empty plasmid) largely reflect the relative transcript levels measured at 37°C for each native locus recombinant strain using qRT-PCR (Fig. 1B). Overall, the differences in *rplT* transcript levels measured at 37°C do not appear to influence or correlate with strain growth at this temperature.

At 15°C, *rplT* transcript levels decreased relative to the *nifU* internal control for all strains during both log and early stationary phase in comparison to the transcript levels quantified at 37°C (Fig. 3B). This is expected, as production of ribosomal components decreases during periods of limited growth and/or under poor growth conditions (Nomura et al. 1984; Bremer and Dennis 2008; Maguire 2009). However, all three regulatory mutant recombinant strains (M1, M2, M4) exhibited an approximately two- to threefold increase in *rplT* levels during log phase at 15°C relative to that of the wild-type recombinant strain, and these elevated transcript levels were considerably more pronounced during early stationary phase at this temperature. Similar to the transcript levels quantified at 37°C, *rplT* transcript in the M3 control mutant recombinant strain was approximately half of that measured in the wild-type recombinant at 15°C. The qRT-PCR data at 15°C suggest that the compromised negative-feedback regulation in the M1, M2, and M4 mutant recombinant strains results in constitutive expression of the *infC* ribosomal protein operon. This expression likely impacts strain growth when rapid ribosome synthesis is not required, such as during entry into stationary phase and the cold temperature conditions assessed in this study.

### L20-interacting RNA mutants demonstrate improper rRNA processing at low temperatures

The cold-sensitive phenotype of the L20-interacting RNA regulatory mutant recombinant strains suggests that removing the RNA-based regulation of the *infC* operon results in aberrant ribosome assembly. To examine if our regulatory mutant recombinant strains are defective in ribosome biosynthesis, we analyzed rRNA 5′ end processing in each strain grown to log phase at both 37°C and 15°C using primer extension. Ribosome assembly and rRNA maturation are tightly coupled. Pre-rRNA transcripts are cleaved to their mature forms concurrent with ribosomal subunit assembly; thus, accumulation of precursor rRNA transcripts and/or improper rRNA processing can be indicative of defects in ribosome composition (Feunteun et al. 1974b; Charollais et al. 2003, 2004; Jain 2008; Choudhury and Flower 2015).

**FIGURE 4.**
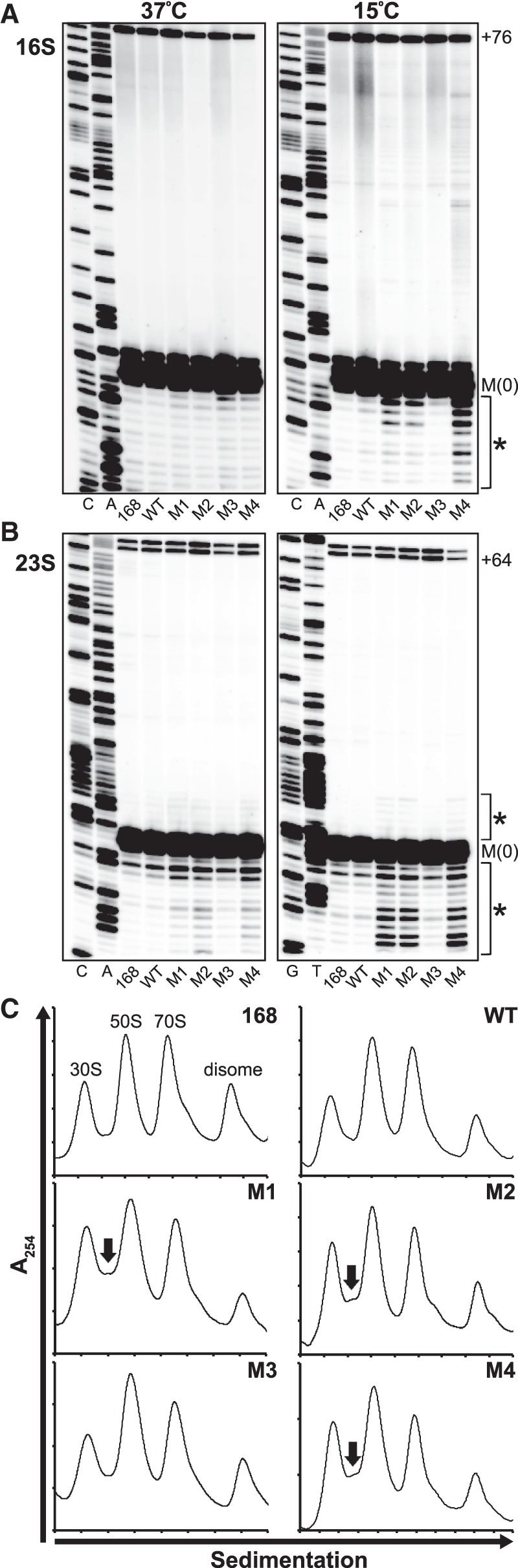
Analysis of ribosome assembly in L20-interacting RNA recombinant strains. Primer extension analysis of 16S rRNA (*A*) and 23S rRNA (*B*) from L20-interacting RNA recombinant strains grown to log phase in 2XYT at 37°C (*left*) and 15°C (*right*). Sequencing reactions performed with the same primers and in vitro transcribed RNAs corresponding to portions of the unprocessed 5′ ends of both the 16S and 23S rRNAs were used to identify the mature 5′ end of each rRNA, labeled as M(0). Sequencing reactions are labeled as their complements to allow for direct reading. Previously characterized precursor species are labeled according to the number of extra nucleotides relative to M(0) and are included for reference (+76 for 16S rRNA, +64 for 23S rRNA) (Britton et al. 2007; Redko et al. 2008). Asterisks (*) indicate regions in which mutant recombinant strain rRNA processing differs from that of the wild-type recombinant strain. Primer extension analysis was conducted with RNA extracted from three or more independent biological replicates of each strain grown to log phase at both 37°C and 15°C. Representative gels are shown. (*C*) Ribosome sedimentation profiles of L20-interacting RNA recombinant strains grown at 15°C. Cells were grown in 2XYT at 15°C with shaking (225 rpm) until an OD_600_ ∼ 0.3–0.4 was reached. Ribosomal subunit sedimentation profiles were resolved by 10%–55% (w/v) sucrose density gradients. Arrows indicate peaks that are present in the profiles of the regulatory mutant recombinant strains, but not in those of the control strains.

Consistent with our previous findings, little to no differences in the 5′ end processing of both the 16S and 23S rRNAs were observed for all recombinant strains when grown at 37°C (Fig. 4A,B). The multiple species of closely spaced mature 5′ ends of each rRNA [indicated as M(0)] have been previously characterized and are due to differences in rRNA sequence across the 10 *rrn* operons encoded within the *B. subtilis* 168 genome, different RNase cleavage sites, and/or other auxiliary processing pathways (Srivastava and Schlessinger 1990; Britton et al. 2007; Redko et al. 2008).

When the strains were grown at 15°C, multiple extension products longer and shorter than those corresponding to the mature 5′ ends of the 16S and 23S rRNAs were apparent in the primer extension reactions of the mutant recombinant strains in which *infC* operon regulation was compromised (M1, M2, M4) (Fig. 4A,B). While the amount of extension product corresponding to the mature 5′ ends of both rRNAs [M(0)] was comparable to those of the 168 parental, wild-type recombinant, and M3 control mutant recombinant strains at this temperature, the extra extension species are likely due to improper rRNA processing brought about by aberrant ribosome composition or an increase in rRNA degradation as a result of the cold-sensitive growth defects previously noted. Cold-sensitive rRNA processing defects have also been observed in bacterial strains lacking specific ribosomal assembly factors (Charollais et al. 2003, 2004; Jain 2008; Bharat and Brown 2014; Choudhury and Flower 2015). None of these additional extension products were present in the 16S and 23S rRNA primer extension reactions of the 168 parental, wild-type recombinant, and M3 control mutant recombinant strains at this temperature. These results are consistent with those from our growth assays and further suggest that disrupting the RNA-based negative-feedback regulation of the *infC* operon leads to defects in ribosome synthesis or assembly.

### Mutations to the L20-interacting RNA affect ribosomal subunit sedimentation

To further characterize the impact disruptions to *infC* operon regulation have on ribosome assembly, we analyzed ribosomal subunit sedimentation profiles of the mutant recombinant strains using sucrose density gradient ultracentrifugation. Because defects in rRNA processing were only apparent in the regulatory mutant recombinant strains during growth at cold temperatures, sedimentation profiles were generated from extracts of cells grown to early log phase at 15°C. For all strains, the peak corresponding to complete 70S ribosomes was reduced relative to that of the 30S and 50S subunits (Fig. 4C). This likely reflects a global reduction in active translation due to suboptimal growth at 15°C (Dai et al. 2016).

Although the ratio of 30S subunits to 50S subunits remained fairly consistent across all strains, a small peak was observed between the 30S and 50S peaks in the sedimentation profiles of the mutant recombinant strains in which the regulatory activity of the L20-interacting RNA was compromised (M1, M2, M4). This peak may correspond to the accumulation of precursor 50S subunits, a population of kinetically trapped “dead-end” particles, intermediates in a pathway for misassembled ribosomal products, or unstable mature ribosomes. Similar peaks have been observed in the sedimentation profiles of bacterial strains in which ribosomal assembly factors have been deleted (Charollais et al. 2003, 2004; Jain 2008) and/or select ribosomal proteins have been overexpressed (Sykes et al. 2010). These data, in combination with the results from our rRNA 5′ end processing assays, indicate that the impact of unregulated *infC* operon expression is formation or accumulation of improper ribosome assembly products.

## DISCUSSION

Many studies have identified, characterized, and compared the structures and mechanisms of the RNA elements responsible for the regulation of ribosomal proteins in bacteria. Furthermore, as the amount of available genomic data continues to increase, additional ribosomal protein *cis*-regulatory RNAs are being discovered, especially in nonmodel species of bacteria. Many of these newly identified regulatory RNA structures are distinct from those previously characterized and are narrowly distributed to select bacterial phyla or classes. Despite these advances in the field, our understanding of the factors that impact the formation, maintenance, and adaptive evolution of these diverse RNA *cis*-regulators within bacterial genomes is relatively nonexistent. To address these questions, we investigated how mutations to the native locus of the L20-interacting RNA *cis*-regulator of ribosomal protein synthesis within the *B. subtilis* genome impact cell fitness. We show that disrupting the regulatory activity of this RNA structure results in constitutive downstream expression and at low temperatures regulatory mutant strains display reduced growth, defects in rRNA processing, and the accumulation of an aberrant ribosome population.

Differences in *rplT* transcript levels, as measured by qRT-PCR, due to mutation and/or misregulation of the *infC* operon do not appear to significantly impact recombinant strain phenotype at 37°C. The M1 and M4 regulatory mutant recombinant strains exhibited an increase in *rplT* levels at this temperature, but both strains grew comparably to the wild-type recombinant at 37°C. Although the M2 regulatory mutant recombinant strain demonstrated *rplT* transcript levels similar to those of the wild-type recombinant during both log and early stationary phase growth at 37°C, the strain grew approximately 1.3 times slower than the wild-type recombinant strain at this temperature. The source of this defect is unclear. One possibility is that there exists higher cell-to-cell variability of *infC* operon expression in the M2 strain that is not obvious from our experiments conducted on bulk culture. However, our microscopy did not reveal more cell size variation for the M2 strain. Despite differences in *rplT* transcript levels and growth at 37°C, all three regulatory mutant recombinant strains exhibited rRNA processing comparable to that of the wild-type recombinant strain at this temperature.

In contrast, expression resulting from the loss of *infC* operon regulation had more severe impacts on regulatory mutant recombinant strain phenotype at 15°C. The M1, M2, and M4 mutant recombinant strains all exhibited elevated constitutive *rplT* transcript levels in comparison to that of the wild-type recombinant at 15°C, and the increased transcript levels were most pronounced during early stationary phase. Subsequently, all regulatory mutant recombinant strains demonstrated similar temperature-sensitive growth, rRNA processing, and ribosome assembly product distribution defects at 15°C.

Unlike the mutations that disrupt the function of the L20-interacting RNA regulator, the M3 control mutation did not cause any mutant phenotypes. Our β-galactosidase assays indicate that the M3 reporter construct retained regulatory activity and the M3 mutant recombinant strain showed no cold-sensitive growth defects and no changes in rRNA processing and ribosomal subunit sedimentation at 15°C compared to the 168 parental and wild-type recombinant strains. Although *rplT* transcript levels in the M3 mutant recombinant strain were lower than that of the wild-type recombinant at both 37°C and 15°C (especially during early stationary phase), the lack of mutant phenotype at both temperatures suggests that this under-expression does not significantly impact the biological outcome in these conditions. The behavior of the M3 control mutant recombinant strain further indicates that the cold-sensitive defects of the L20-interacting RNA M1, M2, and M4 regulatory mutant recombinant strains are primarily the result of unnecessary constitutive expression due to compromised *infC* operon autoregulation. Taken together, these results suggest that RNA *cis*-regulators of ribosomal protein synthesis are important for maintaining a balance between ribosomal protein operon expression and repression, especially under conditions in which the production of ribosomal components is not required, such as during entry into stationary phase or periods of slow or suboptimal growth at low temperatures.

Sensitivity to cold temperatures, reduced growth, rRNA maturation defects, and/or aberrant ribosomal subunit sedimentation profiles are hallmark characteristics of bacterial strains that harbor ribosomal protein mutations or deletions (Guthrie et al. 1969; Tai et al. 1969; Feunteun et al. 1974b; Isono et al. 1976, 1977). These phenotypes have also been observed in bacteria defective in specific rRNA helicases, rRNA or ribosomal protein modification enzymes, rRNA operon copy number, ribosome assembly GTPases, and other assembly and translation cofactors (Charollais et al. 2003, 2004; Jain 2008; Bharat and Brown 2014; Choudhury and Flower 2015; Gyorfy et al. 2015). In this work, we demonstrate that constitutive expression driven by loss of autoregulation from subtle point mutations to a *cis*-regulatory RNA structure within the 5′-UTR of a ribosomal protein operon transcript has similar impacts on ribosome assembly and cell fitness. While most of our evidence points toward ribosome misassembly due to lack of stoichiometry as the root of our cold-sensitive growth defects, it is possible that improper IF3 levels (encoded by *infC*) could also contribute to the observed defects. Our findings reinforce the importance of the coordinated and stoichiometric production of ribosomal components for proper ribosome biosynthesis in bacteria. While the function of RNA *cis*-regulators of ribosomal protein synthesis is well understood, our study highlights the role of these regulatory RNA elements in optimal cell growth and informs us on the selective pressures that influence the formation, evolution, and conservation of structured RNA regulators within bacterial genomes. Understanding the fitness costs associated with the loss of ribosomal protein RNA *cis*-regulators sets the stage for the development of novel antimicrobials that target ribosome synthesis and assembly. RNA *cis*-regulators of ribosomal protein synthesis are a promising antibiotic target, as they are unique to bacteria, almost universal throughout the bacterial world, and are evolved to interact with their protein-binding partners with high specificity.

## MATERIALS AND METHODS

### β-galactosidase activity assay plasmid and strain construction

To generate the protein overexpression plasmid and combat against the growth defects previously noted with strains that solely overexpressed L20 (Choonee et al. 2007), the complete *infC-rpmI-rplT* operon, including the native *infC* Shine-Dalgarno sequence, was PCR-amplified from *B. subtilis* 168 genomic DNA (GenBank: AL009126, complement of 2952224–2953363) (Kunst et al. 1997) with primers containing EcoRI and PstI restriction sites and changing the *infC* ATT start codon to ATG for stronger expression (Supplemental Table S1). After digestion, the PCR product was cloned into the pYH213 plasmid digested with the same enzymes, upstream of a P_T7A1_-*lacO* IPTG-inducible promoter (Yakhnin et al. 2015). This plasmid, as well as an empty control plasmid (pAY132) (Yakhnin et al. 2015), were transformed into *B. subtilis* 168 as described previously (Yasbin et al. 1975). Transformants were screened for resistance to tetracycline (12.5 µg/mL) and verified via PCR.

To generate the *infC*’-’*lacZ* reporter constructs, the region containing the second native *infC* operon promoter (the major promoter for *infC* expression [Choonee et al. 2007; Bruscella et al. 2011]), the wild-type L20-interacting RNA leader, and Shine-Dalgarno sequence and first nine codons of *infC* was PCR-amplified from *B. subtilis* 168 genomic DNA (GenBank: AL009126, complement of 2953323–2953586) with primers containing EcoRI and BamHI restriction sites and changing the *infC* ATT start codon to ATG for stronger expression (Supplemental Table S1). The PCR product was cloned in-frame as a translational fusion with a *lacZ* reporter into a modified pDG1728 plasmid digested with the same enzymes (Guérout-Fleury et al. 1996; Babina et al. 2017). Mutations to the L20-interacting RNA were obtained by site-directed mutagenesis or PCR assembly and verified via Sanger sequencing (Supplemental Table S1). Reporter constructs were transformed into the above *B. subtilis* 168 pYH213/pAY132 protein overexpression strains as described previously (Jarmer et al. 2002). Transformants were selected on TBAB + 12.5 µg/mL tetracycline + 100 µg/mL spectinomycin and screened for proper integration of the *lacZ* reporter constructs into the *amyE* locus based on sensitivity to erythromycin (0.5 µg/mL) and loss of amylase activity (plating on TBAB + 1% starch and staining with Gram's iodine solution, Sigma-Aldrich).

### β-galactosidase activity assays

*B. subtilis* 168 *lacZ* reporter strains were grown from single colonies in 2 mL 2XYT + 12.5 µg/mL tetracycline + 100 µg/mL spectinomycin for 16–18 h at 37°C with shaking (225 rpm). These cultures (30 µL for the pAY132 strains, 50 µL for the pYH213*-infC-rpmI-rplT* strains) were used to inoculate 2 mL 2XYT + 12.5 µg/mL tetracycline + 100 µg/mL spectinomycin + 1 mM IPTG cultures, which were then grown at 37°C with shaking (225 rpm) until an OD_600_ ∼ 0.3–0.7 was reached. Cells (1 mL) were harvested and resuspended in 1 mL Z buffer (50 mM Na_2_HPO_4_, 40 mM NaH_2_PO_4_, 10 mM KCl, 1 mM MgSO_4_, 50 mM 2-Mercaptoethanol) + 100 µg/mL spectinomycin. β-galactosidase activity assays were performed as previously described using 0.05 mL of cell suspensions. Miller Units were calculated as follows (Miller 1992):

$$\text{Miller Units}=1000*\frac{A_{420}}{\Delta t(\min)*A_{600}*vol.(\mathrm{mL})}.$$

The values reported represent the mean of three or more independent replicates; error bars represent standard error of the mean across biological replicates. To determine the fold repression for each RNA reporter construct, the mean Miller Units for each empty plasmid reporter strain was divided by that of the corresponding *infC* operon overexpression strain. Standard error for the fold repression values were propagated from the error calculated for the Miller Units as described previously (Taylor 1997).

### L20-interacting RNA native locus recombinant strain construction

The recombinant strains for the growth assays, qRT-PCR, rRNA processing assays, and ribosome sedimentation profiles were generated as described previously (Babina et al. 2017). Briefly, the second *infC* operon promoter, wild-type L20-interacting RNA leader, and two ∼500 bp regions of homology flanking the promoter and regulatory RNA leader region were PCR-amplified from *B. subtilis* 168 genomic DNA (GenBank: AL009126; complement of 2953587–2954227 for the 5′ flanking ∼500 bp region of homology, complement of 2953142–2953586 for the region containing the promoter, RNA leader, and 3′ flanking ∼500 bp region of homology). A PCR product in which an erythromycin resistance cassette was introduced into the intergenic region immediately upstream of the second *infC* operon promoter was generated and cloned into pCR2.1 or pCR4 TOPO-TA vector (Invitrogen). Mutations to the L20-interacting RNA were obtained via site-directed mutagenesis or PCR assembly (Supplemental Table S1). These constructs were then transformed into *B. subtilis* 168 as described previously and transformants were screened for resistance to erythromycin (0.5 µg/mL) (Jarmer et al. 2002). Integration of the complete recombinant construct within the *infC* locus and the presence of the L20-interacting RNA mutations of interest were verified via PCR and Sanger sequencing.

### Growth assays

For the growth assays at 37°C, *B. subtilis* 168 strains were grown from single colonies in 0.5 mL 2XYT (+0.5 µg/mL erythromycin for recombinant strains) in sterile nontreated 24-well cell culture plates for 16–18 h at 37°C with shaking (225 rpm). These cultures were used to inoculate 0.5 mL 2XYT (+0.5 µg/mL erythromycin for recombinant strains) cultures to a starting OD_600_ ∼ 0.05 in sterile nontreated 24-well cell culture plates. Plates were incubated at 37°C with shaking (225 rpm) for ∼24 h. OD_600_ values were recorded at time points indicated using a SpectraMax M3 Multi-Mode Microplate Reader (Molecular Devices).

For the growth assays at 15°C*, B. subtilis* 168 strains were grown from single colonies in 2 mL 2XYT (+0.5 µg/mL erythromycin for recombinant strains) for 16–18 h at 37°C with shaking (225 rpm). Because cultures were not viable in 24-well plates for an extended period of time at 15°C, these cultures were used to inoculate 25 mL 2XYT (+0.5 µg/mL erythromycin for recombinant strains) cultures to a starting OD_600_ ∼ 0.05 in sterile 250 mL flasks. Flasks were incubated at 15°C with shaking (225 rpm) for ∼100 h. OD_600_ values were recorded at time points indicated using a NanoDrop 2000c (Thermo Fisher Scientific).

Doubling times were calculated as previously described using log phase log_10_(OD_600_) values (Rubinow 1975). Briefly, doubling time is the inverse of the slope *m* determined from log_10_(OD_600_) versus time graphs during log phase growth, multiplied by 0.301 [corresponds to ln(2)/ln(10)]. The values reported represent the mean of three or more independent replicates; the error reported is the standard error of the mean across biological replicates. To determine the significance, mutant recombinant strain doubling times were compared to those of the wild-type recombinant strain at the corresponding temperature using a Welch's single-tailed *T*-test in Microsoft Excel. Values were considered significantly different if *P* < 0.05. Representative growth curves are shown.

### Cell size measurements

*B. subtilis* 168 strains were grown to log phase (OD_600_ ∼ 0.4–0.5) in 2XYT (+0.5 µg/mL erythromycin for recombinant strains) at 37°C and 15°C with shaking (225 rpm) as described above. Cells were washed and resuspended in 1× phosphate-buffered saline (137 mM NaCl, 2.7 mM KCl, 10 mM Na_2_HPO_4_, 1.8 mM KH_2_PO_4_, pH 7.4), and imaged using DIC on a Zeiss AxioImager Z2 microscope with a Plan-Apochromat 63×/1.40 objective and a Hamamatsu ORCA-R2 CCD camera. Measurements were made directly from images using FIJI software (Schindelin et al. 2012). The lengths and widths of 100 cells from each strain were measured and averaged at each temperature; the error reported is the standard error of the mean. Image files were renamed and randomized prior to analysis to prevent bias. To determine the significance, mutant recombinant strain cell length and width were compared to those of the wild-type recombinant strain at the corresponding temperature using a Welch's single-tailed *T*-test in Microsoft Excel. Values were considered significantly different if *P* < 0.05.

### Quantitative RT-PCR

Total RNA was extracted from early-to-mid log (OD_600_ ∼ 0.3–0.7) and early stationary phase (OD_600_ ∼ 2.0–3.0) *B. subtilis* 168 cultures grown in 20 mL 2XYT (+0.5 µg/mL erythromycin for recombinant strains) at both 37°C and 15°C with shaking (225 rpm). Genomic DNA was removed from 5 µg of total RNA by digestion with RQ1 DNase (Promega) at 37°C for 40 min, followed by heat inactivation at 98°C for 2 min, phenol–chloroform extraction, and ethanol precipitation. Reverse transcription was performed using the DNase-treated RNA, random hexamer, and SuperScript III according to the manufacturer's protocol (Invitrogen). qPCR was conducted with the resulting cDNA using an ABI 7500 Fast Real-Time PCR system and SYBR green detection (ThermoFisher Scientific). *infC* operon transcript expression was quantified using primers targeting the *rplT* coding region and expression of *nifU* was used as an internal normalization control (Supplemental Table S1; Reiter et al. 2011). Experiments were repeated using reactions lacking reverse transcriptase to confirm removal of genomic DNA. Error bars represent the standard error of the mean across three technical replicates propagated using previously described calculations (Taylor 1997).

### Primer extension assays

Log phase total RNA was extracted from *B. subtilis* 168 cultures grown at both 37°C and 15°C as described above. For sequencing reaction templates, regions corresponding to the unprocessed 5′ ends of both the 16S and 23S rRNAs from the *rrnW* operon were PCR-amplified from *B. subtilis* 168 genomic DNA using forward primers that included the T7 promoter sequence (Supplemental Table S1). PCR products were gel-purified, cloned into pCR2.1 TOPO-TA vector (Invitrogen) for sequencing, and RNA was transcribed from these constructs using T7 RNA polymerase and purified by 6% denaturing PAGE (Milligan et al. 1987). Synthetic oligonucleotide DNA primers (20 pmol, IDT) complementary to the mature 5′ ends of the 16S and 23S rRNAs were 5′-end labeled with [γ-^32^P] ATP (PerkinElmer) and purified via 12% denaturing PAGE (Supplemental Table S1) (Regulski and Breaker 2008).

For the primer extension reactions, 5 µg of total RNA or 1 pmol of in vitro transcribed RNA was combined with 1 µL of ^32^P-labeled primer (∼30,000–50,000 cpm/µL) for a final volume of 12 µL in water. This mixture was denatured at 75°C for 4 min and then flash frozen in a dry ice/ethanol bath for 2 min before being transferred to ice. SuperScript III (1 µL/200 U, Invitrogen) and 7 µL of a master mix were added to each reaction, for a total reaction volume of 20 µL and a final concentration of 50 mM Tris HCl [pH 8.3], 75 mM KCl, 3 mM MgCl_2_, 5 mM DTT, 0.5 mM each dNTP, and 20 U SUPERase-In (Invitrogen). For the sequencing reactions, 1 µL of 100 mM ddNTP (TriLink Biotechnologies) was also added to the appropriate reaction. Reactions were incubated at 55°C for 30 min, stopped with 20 µL of formamide loading dye (95% formamide, 20 mM EDTA [pH 8.0], 0.05% bromophenol blue, 0.05% xylene cyanol), and 8 µL of each sample was separated by 10% denaturing PAGE. Prior to loading, samples were heated at 75°C for 4 min and then cooled on ice for 2 min. Gels were dried, exposed to phosphor screens for 48–72 h, and imaged using a Typhoon FLA 9500 scanner (GE Life Sciences) (modified from Britton et al. 2007). Primer extension reactions were performed on RNA extracted from three or more independent biological replicates of each strain, grown to log phase at both 37°C and 15°C. Representative gels are shown.

### Ribosome sedimentation profiles

*B. subtilis* 168 strains were grown from single colonies in 2 mL 2XYT (+0.5 µg/mL erythromycin for recombinant strains) for ∼16–18 h at 37°C with shaking (225 rpm). These cultures were used to inoculate 250 mL 2XYT (+0.5 µg/mL erythromycin for recombinant strains) cultures to a starting OD_600_ ∼ 0.05 in sterile 1 L flasks. Flasks were incubated at 15°C with shaking (225 rpm) until an OD_600_ ∼ 0.3–0.4 was reached. Samples were prepared as previously described (Li et al. 2014). Briefly, cells were harvested by rapid filtration using BioTrace NT pure nitrocellulose 0.2 µm membrane filters (Pall Life Sciences) and flash frozen in liquid nitrogen. Cell pellets were combined with 550 µL of frozen droplets of lysis buffer (20 mM Tris [pH 8.0], 100 mM NH_4_Cl, 10 mM MgCl_2_, 0.4% Triton X-100, 0.1% NP-40, 1 mM chloramphenicol, 100 U/mL DNase I) in 10 mL canisters (Retsch) prechilled in liquid nitrogen and pulverized by mixer milling (Qiagen Tissuelyzer II). The resulting lysates were thawed and clarified by centrifugation, and 200 µL of the supernatants were layered onto 10%–55% (w/v) sucrose gradients prepared in 20 mM Tris [pH 8.0], 100 mM NH_4_Cl, 10 mM MgCl_2_, and 1 mM chloramphenicol. Gradients were centrifuged at 35000 rpm at 4°C for 2.5 h (Beckman Coulter) and then analyzed at 254 nm using a Biocomp Gradient Station iP, Bio-Rad Econo UV Monitor, and Gradient Profiler software.

## SUPPLEMENTAL MATERIAL

Supplemental material is available for this article.

## Supplementary Material

Supplemental Material
