# Supplementary material for: Fitness advantages conferred by the L20-interacting RNA cis-regulator of ribosomal protein synthesis in Bacillus subtilis
Source: RNA. 2018 Sep;24(9):1133–43. doi: 10.1261/rna.065011.117 (PMC6097659; doi:10.1261/rna.065011.117)
Supplement: Supplemental Material [file supp_24_9_1133__index.html]

Fitness advantages conferred by the L20-interacting RNA cis-regulator of ribosomal protein synthesis in Bacillus subtilis — Supplemental Material 

# Fitness advantages conferred by the L20-interacting RNA *cis*-regulator of ribosomal protein synthesis in *Bacillus subtilis*

## Supplemental Material

- Supplemental\_Material.pdf
