# Supplementary material for: Fitness advantages conferred by the L20-interacting RNA cis-regulator of ribosomal protein synthesis in Bacillus subtilis
Source: RNA. 2018 Sep;24(9):1133–43. doi: 10.1261/rna.065011.117 (PMC6097659; doi:10.1261/rna.065011.117)
Supplement: Supplemental Material [file supp_065011.117_Supplemental_Material.pdf]

**SUPPLEMENTAL MATERIAL FOR:**

# Fitness advantages conferred by the L20-interacting RNA *cis*-regulator of ribosomal protein synthesis in *Bacillus subtilis*

Arianne M. Babina,[1,3] Darren J. Parker,[2] Gene-Wei Li,[2] and Michelle M. Meyer[1,*]

[1]Department of Biology, Boston College, Chestnut Hill, Massachusetts 02467, USA

[2]Department of Biology, Massachusetts Institute of Technology, Cambridge, Massachusetts 02139, USA

[3]Present address: Department of Medical Biochemistry and Microbiology, Uppsala University, 752 37 Uppsala, Sweden

[*]Corresponding author: m.meyer@bc.edu

**Running title:** Fitness benefits of ribosomal protein autoregulation

**This PDF includes:**
Materials and Methods
Figure S1
Table S1
References

## MATERIALS AND METHODS

### Re-designed L20-interacting RNA native locus recombinant strain construction

To generate recombinant L20-interacting RNA strains in which the first native *infC* promoter immediately precedes the second *infC* promoter, new 5’ and 3’ ~500 bp regions of homology that flank the *infC* locus were PCR-amplified from *B. subtilis* 168 genomic DNA as described (GenBank: AL009126; complement of 2953643-2954227 for the 5’ flanking ~500 bp region of homology, complement of 2953142-2953642 for the region containing the promoters, regulatory RNA, and 3’ flanking ~500 bp region of homology). To prevent read-through from the erythromycin resistance cassette promoter, the region containing two rho-independent terminators was PCR-amplified from the pYH213 plasmid (Yakhnin et al. 2015) and appended onto the 3’ end of the erythromycin resistance cassette via PCR assembly. Recombinant constructs were assembled and transformed into *B. subtilis* 168 as described previously (Babina et al. 2017).

### Northern blots

Total RNA was extracted from select recombinant *B. subtilis* 168 strains grown to log phase at 37ºC as described. As a size-standard, template DNA corresponding to both the full-length and terminated *infC-rpmI-rplT* operon transcripts were PCR-amplified from *B. subtilis* 168 genomic DNA using forward primers that contained the T7 promoter sequence (GenBank: AL009126; complement of 2952168-2953550 for the full-length transcript, complement of 2953394-2953550 for the terminated transcript) (Table S1). T7 RNA polymerase was used to transcribe RNA from these templates, and transcription reactions were ethanol precipitated and re-suspended in TE buffer (Milligan et al. 1987). These transcripts (15 ng of full-length transcript, 3 ng of terminated transcript) and 10 $\mu$g of each total RNA sample were separated on a 0.8% denaturing agarose gel and transferred to an Amersham Hybond-N+ membrane (GE Healthcare) overnight, as described previously (Rio 2015). The membrane was UV-crosslinked and pre-hybridized in 10 mL Ambion ULTRAhyb ultrasensitive hybridization buffer (Life Technologies) at 37ºC or 42ºC for ~2-4 hours with rotation. Synthetic oligonucleotide DNA probes (40 pmol, IDT) complementary to transcript regions of interest were 5’-end labeled with [$\gamma$-$^{32}$P] ATP (Perkin Elmer) (Regulski and Breaker 2008) and allowed to hybridize to the membrane overnight at 37ºC or 42ºC with rotation (Table S1). The membrane was washed twice with approximately 100 mL 2X SSC (300 mM NaCl, 30 mM trisodium citrate [pH 7.0]) + 0.1% SDS at 37ºC or 42ºC for 20 minutes with rotation, exposed to a phosphor screen for 16-72 hours, and imaged using a Typhoon FLA 9500 scanner (GE Life Sciences). The membrane was then stripped with boiling hot water + 0.1% SDS at 70ºC with rotation until signal was removed and re-probed for a different transcript as described above.

### 5’-RACE

Total RNA was extracted from log phase wild-type recombinant *B. subtilis* 168 cultures grown at 37ºC as previously described and 5’-RLM-RACE was performed following the Invitrogen GeneRacer protocol with a homemade RNA-linker (Weinberg et al. 2009). First-strand synthesis was performed with a gene-specific primer complementary to the erythromycin resistance cassette coding region (primer 1401, Table S1). The resulting cDNA was used as template for PCR with a forward primer specific to the RNA-linker and a nested reverse primer specific to the erythromycin resistance cassette coding region (primers 9 and 1400, respectively, Table S1). PCR products were cloned into pCR2.1 TOPO-TA vector (Invitrogen) and sequenced to identify transcription start sites.

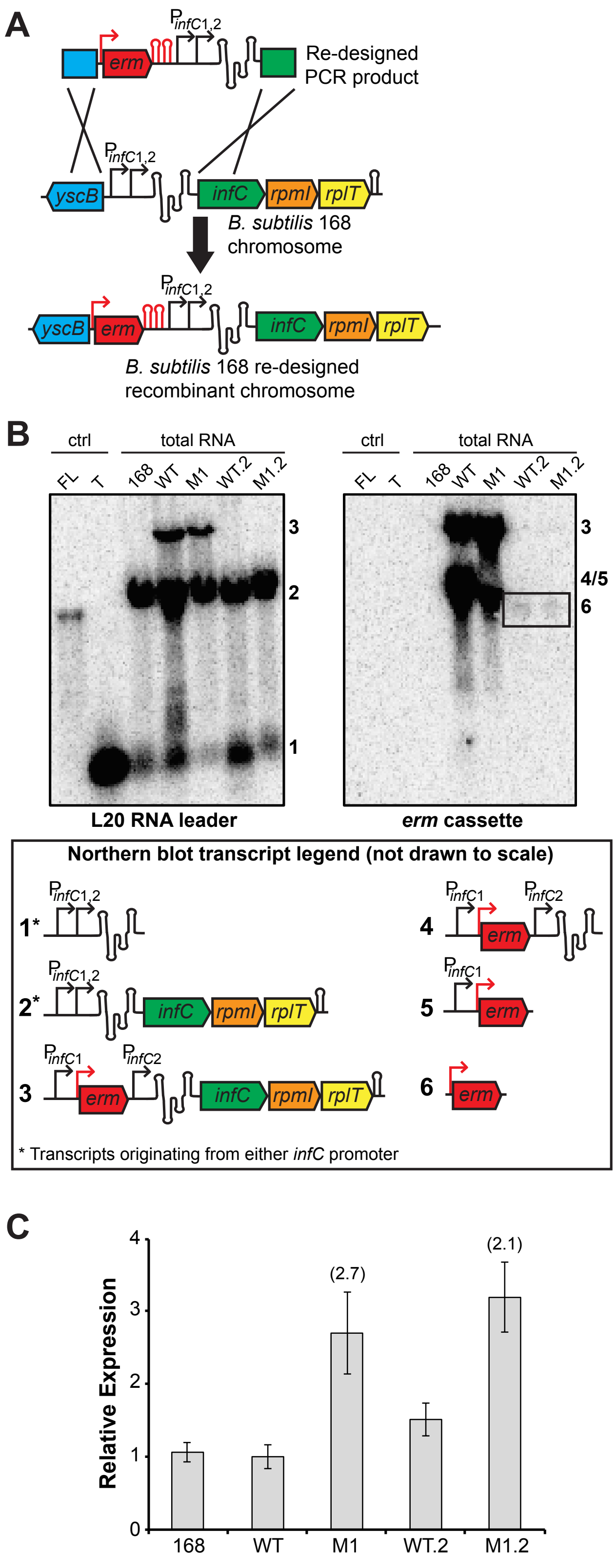


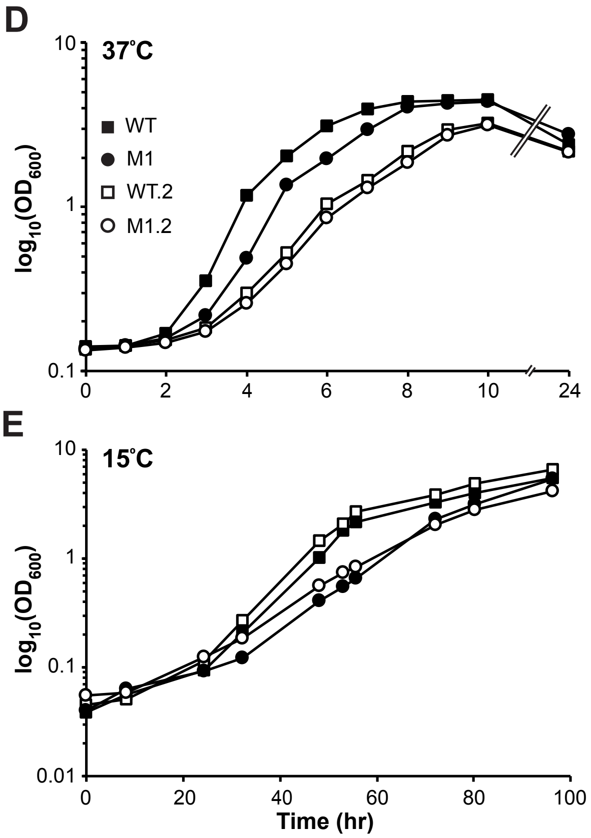


F

**Doubling Time (min)**

| Strain | 37ºC | 15ºC |
|---|---|---|
| **168** | 53 ± 1 | 346 ± 4 |
| **WT** | 68 ± 2 | 385 ± 3 |
| **M1** | 69 ± 1 (1.0) | 538 ± 31 (1.4)^ |
| **WT.2** | 86 ± 1 | 414 ± 22 |
| **M1.2** | 89 ± 1 (1.0)* | 720 ± 23 (1.7)# |

* p = 0.048
^ p = 0.019
# p = 0.005

**Figure S1.** *Legend on following page.*

**Figure S1.** Construction and confirmation of the re-designed L20-interacting RNA recombinant strains (WT.2, M1.2). (A) Re-designed L20-interacting RNA recombinant strain constructs. New PCR products were constructed to preserve the native organization of the two *infC* operon promoters. Re-designed recombinant strains were generated as described in Fig. 2A, except the *erm* cassette was introduced into the intergenic region immediately upstream of the first *infC* operon promoter and two transcription terminators were appended onto the 3' end of the *erm* cassette to prevent potential read-through from the *erm* promoter. (B) Northern blot analysis of total log phase RNA from original (WT, M1) and re-designed (WT.2, M1.2) recombinant L20-interacting RNA strains grown in 2XYT at 37ºC. The membrane was hybridized with oligonucleotide probes complementary to the L20-interacting RNA leader (left) and the erythromycin (*erm*) resistance cassette (right) (Table S1). *In vitro* transcribed RNAs corresponding to the full length (ctrl FL) and terminated (ctrl T) *infC-rpmI-rplT* operon transcripts were included as size standards. Transcript resulting from read-through from the displaced first *infC* operon promoter and/or the *erm* cassette promoter (Northern blot transcript legend, 3) is apparent in the original L20-interacting RNA recombinant strains and was confirmed using 5'-RACE. This transcript is not present in the re-designed recombinant strains due to restoration of the native *infC* operon promoter organization and the introduction of two terminators at the end of the *erm* cassette coding sequence. Transcripts 4 and 5 (see Northern blot transcript legend) are potential transcripts that cannot be distinguished on the blots shown; transcript 4 may also be present in the original recombinant strains (WT, M1) on the L20 RNA leader blot (left). Transcription from the displaced first *infC* promoter greatly increased *erm* expression in the original recombinant strains relative to that of the re-designed recombinant strains. (C) qRT-PCR quantification of the native *rplT* transcript in the re-designed recombinant strains (WT.2, M1.2) grown to log phase at 37ºC. *rplT* transcript level was normalized to the level of housekeeping gene *nifU* (Reiter et al. 2011). Graphs depict relative *rplT* transcript levels in each strain compared to the *rplT* level in the original wild-type recombinant (WT) strain grown to log phase at 37ºC. Error bars represent the standard error of the mean across three technical replicates. Numbers in parentheses denote mutant recombinant strain *rplT* transcript level relative to that of the corresponding wild-type recombinant strain. Consistent with the qRT-PCR results from our original recombinant strains, the re-designed M1.2 recombinant strain exhibited elevated *rplT* transcript levels relative to that of the re-designed WT.2 recombinant strain. (D,E) Comparison of original (WT, M1) and re-designed (WT.2, M1.2) L20-interacting RNA recombinant strain growth. Growth curves for each recombinant strain grown in 2XYT at 37ºC (D) and 15ºC (E). Growth assays were performed two or more times for each strain. Representative curves are shown. (F) Doubling times (min) of original and re-designed L20-interacting RNA recombinant strains grown in 2XYT at 37ºC and 15ºC. Values were calculated from log phase $OD_{600}$ values and are the mean of two or more independent experimental replicates; ± indicates the standard error of the mean across biological replicates. Numbers in parentheses denote mutant recombinant strain doubling time relative to that of the appropriate wild-type recombinant (WT or WT.2) strain at the corresponding temperature. Symbols (*,^,#) indicate mutant recombinant strains that grew significantly slower than the appropriate wild-type recombinant at the corresponding temperature; the p values are indicated in the figure.

**Table S1.** Oligonucleotides used in this study.
For primer pairs, the forward primer is listed first and the reverse primer is listed second.

| Name | Sequence (5'-3') | Notes |
|---|---|---|
| 1349 | GGCCC**GAATTC**TATGGAGGTGGCTCATGATTAGCAAAGATC | Primer for cloning *infC-rpmI-rplT* operon into pYH213 protein overexpression plasmid (Yakhnin et al. 2015); EcoRI restriction site in bold; ATT to ATG is underlined |
| 1351 | GCCGG**CTGCAG**TTACTTGTTTAATTGAG | Primer for cloning *infC-rpmI-rplT* operon into pYH213 protein overexpression plasmid (Yakhnin et al. 2015); PstI restriction site in bold |
| 1357 | GTTGATCAGTCAACTTATCTGTATAG | Primer for PCR checks and sequencing of pYH213 protein overexpression constructs |
| 1358 | CGTTTAAGGGCACCAATAACTG | Primer for PCR checks and sequencing of pYH213 protein overexpression constructs |
| 866 | CGCGC**GAATTC**TTGACTAAAGATCCGGTATTGTGTAG | Primer for cloning L20-interacting RNA into modified pDG1728 reporter plasmid (Babina et al. 2017); EcoRI restriction site in bold |
| 867 | GCGC**GGATCC**ATTAACCAATTGATCTTTGCTAATCAT | Primer for cloning L20-interacting RNA into modified pDG1728 reporter plasmid (Babina et al. 2017); BamHI restriction site in bold; ATT to ATG is underlined |
| 204 | TATCTCTTGCCAGTCACGTTACG | Primer for PCR checks and sequencing of pDG1728 reporter constructs |
| 122 | GGGGACGACGACAGTATCGGCCTC | Primer for PCR checks and sequencing of pDG1728 reporter constructs |
| 691 | CGA**GGATCC**TCGCTTTCTGCTCTTTTTGGATTG | Primer for amplifying 5'-500 bp region of L20-interacting RNA recombinant construct; BamHI restriction site in bold, if needed |
| 692 | CTTTAGGGTTATCGAATTCGATAAGCTTCTAGGACGACTTATCCGGAACAACTTTTACATTG | Primer for amplifying 5'-500 bp region of L20-interacting RNA recombinant construct |
| 693 | CCCTAGCGCCTACGGGGAATTTGTATCGCGGCCGCTTGACTAAAGATCCGGTATTGTGTAG | Primer for amplifying 3'-500 bp region of L20-interacting RNA recombinant construct |
| 774 | GCTCAAATCGGAACTTACCGTAG | Primer for amplifying 3'-500 bp region of L20-interacting RNA recombinant construct |
| 681 | TAGAAGCTTATCGAATTCGATAACCCTAAAG | Primer for amplifying erythromycin resistance cassette from pDG1663 (Guérot-Fluery et al. 1996) |

| | | |
|---|---|---|
| 682 | GCGGCCGCGATACAAATTAAAAGTAGGCG | Primer for amplifying erythromycin resistance cassette from pDG1663 (Guérot-Fluery et al. 1996) |
| 720 | CGTCATCAGGAAGCTCTGAAGCTG | Primer for confirming genomic integration of recombinant constructs |
| 775 | GAATTTAATCGCATTGCGCAATTTCGTG | Primer for confirming genomic integration of recombinant constructs |
| 745 | GCAATGAAACACGCCAAAGTAAAC | Primer for PCR checks, sequencing recombinant L20-interacting RNA constructs |
| 1081 | GAATTGAATATAAATCACTTGCAGAAGCACC CGCTTC | Mutagenesis primer for M1, protein-binding mutation |
| 1082 | GAAGCGGGTGCTTCTGCAAGTGATTTATATT CAATTC | Mutagenesis primer for M1, protein-binding mutation |
| 746 | GAAGCACCCGCTTCTCAGGTGATTGACACA TGC | Mutagenesis primer for M2, protein-binding mutation |
| 747 | GCATGTGTCAATCACCTGAGAAGCGGGTGC TTC | Mutagenesis primer for M2, protein-binding mutation |
| 1294 | GACCGTACATTTTTACCGATACAGATGTTCG TAG | Mutagenesis primer for M3, control mutation |
| 1295 | CTACGAACATCTGTATCGGTAAAAATGTACG GTC | Mutagenesis primer for M3, control mutation |
| 1412 | GGGTGTTTTATAATGCGGTGCATTTTTGTTT GCCTGC | Mutagenesis primer for M4, terminator mutation |
| 1413 | GCAGGCAAACAAAAATGCACCGCATTATAAA ACACCC | Mutagenesis primer for M4, terminator mutation |
| 1692 | GGAAACTATGCTTTCCGTGACC | Primer for qRT-PCR targeting the *rplT* coding region |
| 1693 | TAAGAAAGGCCGTTCATGCG | Primer for qRT-PCR targeting the *rplT* coding region |
| 1546 | TTTTACTTCGTGACGGCGGT | Primer for qRT-PCR targeting *nifU*, the normalization control |
| 1547 | TTGTTGAACTTGGGCAGCTG | Primer for qRT-PCR targeting *nifU*, the normalization control |
| 8 | TAATACGACTCACTATAGG | T7 promoter primer for generating 5'-RACE RNA-linker |
| 7 | TTTCTACTCCTTCAGTCCATGTCAGTGTCCT CGTGCTCCAGTCGCCTATAGTGAGTCGTATT A | Primer for generating 5'-RACE RNA-linker |
| 1401 | CAGATAGATGTCAGACGCATGGC | Erythromycin resistance cassette outer reverse primer for cDNA synthesis (RT) |
| 9 | GACTGGAGCACGAGGACACTGA | 5'-RACE RNA-linker forward primer for PCR |
| 1400 | GCCAGTTTCGTCGTTAAATGCCC | Erythromycin resistance cassette nested reverse primer for PCR |

| | | |
|---|---|---|
| 1107 | GCGGGTGCTTCTGCTTGTGATTTATAT | Northern blot probe for L20-interacting RNA (Choonee et al. 2007); hybridize and wash at 42ºC |
| 1402 | GTTTACTTTGGCGTGTTTCATTGC | Northern blot probe for erythromycin resistance cassette coding sequence; hybridize and wash at 37ºC |
| 1448 | CTTTAGGGTTATCGAATTCGATAAGCTTCTAGAGAGAAAAGAAAATCTTTCATCCCCAC | Primer for amplifying 5'-500 bp region of L20-interacting RNA re-designed recombinant construct; use with 691 |
| 1449 | CGCCTACGGGGAATTTGTATCGTTTAAACTTGCGCTCATAGAAAACCCATGTTACAATG | Primer for amplifying 3'-500 bp region of L20-interacting RNA re-designed recombinant construct; use with 774 |
| 1444 | CTGACAGCTTCCAAGGAGCTAAAGAGGTCTCCTGTTGATAGATCCAGTAATGACC | Primer for amplifying double terminator construct from pYH213 (Yakhnin et al. 2015) for appending onto 3’ end of erythromycin resistance cassette from pDG1663 for re-designed recombinant constructs |
| 1445 | GGTCATTACTGGATCTATCAACAGGAGACCTCTTTAGCTCCTTGGAAGCTGTCAG | Primer for amplifying double terminator construct from pYH213 (Yakhnin et al. 2015) for appending onto 3’ end of erythromycin resistance cassette from pDG1663 for re-designed recombinant constructs |
| 1446 | GTTTAAACGATACAAATTCCCCGTAGGCGCTAGGGAAAAAAATTACGCCCCGCCCTGCC | Primer for appending double terminator construct onto 3’ end of erythromycin resistance cassette from pDG1663 for re-designed recombinant constructs; use with primer 681 |
| 1109 | <u>CCAAGTAATACGACTCACTATAG</u>GAATTGAATATAAATCACAAGCAGAAG | T7 *in vitro* transcription primer for *infC* operon transcription start; T7 promoter sequence is underlined |
| 1110 | GAATGCATTTTGCAGGC | T7 *in vitro* transcription primer for terminated *infC* operon transcript |
| 1296 | GTTGCCTCATCCTTTATATAG | T7 *in vitro* transcription primer for full-length *infC* operon transcript |
| 1621 | <u>CCAAGTAATACGACTCACTATAG</u>GCTTTAACAAAGCGGACAAACAAAATGATC | T7 *in vitro* transcription primer for 16S rRNA sequencing reaction template (modified from Britton et al. 2007); T7 promoter sequence is underlined |

| | | |
|---|---|---|
| 1432 | CAGCGTTCGTCCTGAGCCAG | Primer for 16S rRNA sequencing reaction template, primer extension reactions (modified from Britton et al. 2007) |
| 1623 | CCAAGTAATACGACTCACTATAGGACCTTGG GTCTTATAAACAGAACG | T7 *in vitro* transcription primer for 23S rRNA sequencing reaction template (modified from Redko et al. 2008); T7 promoter sequence is underlined |
| 1434 | CATCGGCTCCTAGTGCCAAGGCATC | Primer for 23S rRNA sequencing reaction template, primer extension reactions (modified from Redko et al. 2008) |

## REFERENCES

Babina AM, Lea NE, Meyer MM. 2017. *In Vivo* Behavior of the Tandem Glycine Riboswitch in *Bacillus subtilis*. *mBio* **8**: e01602–17.

Britton RA, Wen T, Schaefer L, Pellegrini O, Uicker WC, Mathy N, Tobin C, Daou R, Szyk J, Condon C. 2007. Maturation of the 5' end of *Bacillus subtilis* 16S rRNA by the essential ribonuclease YkqC/RNase J1. *Molecular Microbiology* **63**: 127–138.

Choonee N, Even S, Zig L, Putzer H. 2007. Ribosomal protein L20 controls expression of the *Bacillus subtilis infC* operon via a transcription attenuation mechanism. *Nucleic Acids Res* **35**: 1578–1588.

Guérout-Fleury A, Frandsen N, Stragier P. 1996. Plasmids for ectopic integration in *Bacillus subtilis*. *Gene* **180**: 57–61.

Milligan J, Groebe D, Witherell G, Uhlenbeck O. 1987. Oligoribonucleotide synthesis using T7 RNA polymerase and synthetic DNA templates. *Nucleic Acids Res* **15**: 8783–8798.

Redko Y, Bechhofer DH, Condon C. 2008. Mini-III, an unusual member of the RNase III family of enzymes, catalyses 23S ribosomal RNA maturation in *B. subtilis*. *Molecular Microbiology* **68**: 1096–1106.

Regulski EE, Breaker RR. 2008. In-line probing analysis of riboswitches. *Methods Mol Biol* **419**: 53–67.

Reiter L, Kolstø A-B, Piehler AP. 2011. Reference genes for quantitative, reverse-transcription PCR in *Bacillus cereus* group strains throughout the bacterial life cycle. *J Microbiol Methods* **86**: 210–217.

Rio DC. 2015. Northern blots: Capillary transfer of RNA from agarose gels and filter hybridization using standard stringency conditions. *Cold Spring Harb Protoc* 306–313.

Weinberg Z, Perreault J, Meyer MM, Breaker RR. 2009. Exceptional structured noncoding RNAs revealed by bacterial metagenome analysis. *Nature* **462**: 656–659.

Yakhnin H, Yakhnin AV, Babitzke P. 2015. Ribosomal protein L10(L12)$_4$ autoregulates expression of the *Bacillus subtilis rplJL* operon by a transcription attenuation mechanism. *Nucleic Acids Res* **43**: 7032–7043.
